# An Online Pre-procedural Nomogram for the Prediction of Contrast-Associated Acute Kidney Injury in Patients Undergoing Coronary Angiography

**DOI:** 10.3389/fmed.2022.839856

**Published:** 2022-03-11

**Authors:** Duanbin Li, Hangpan Jiang, Xinrui Yang, Maoning Lin, Menghan Gao, Zhezhe Chen, Guosheng Fu, Dongwu Lai, Wenbin Zhang

**Affiliations:** ^1^Department of Cardiology, Sir Run Run Shaw Hospital, College of Medicine, Zhejiang University, Hangzhou, China; ^2^Key Laboratory of Cardiovascular Intervention and Regenerative Medicine of Zhejiang Province, Hangzhou, China; ^3^Department of Cardiology, The Fourth Affiliated Hospital, College of Medicine, Zhejiang University, Yiwu, China; ^4^Department of Cardiology, The Hangzhou Lin’an People’s Hospital, Hangzhou, China; ^5^College of Medicine, Zhejiang University, Hangzhou, China

**Keywords:** contrast-associated acute kidney injury, nomogram, coronary artery disease, coronary angiography, percutaneous coronary intervention

## Abstract

**Background:**

Identifying high-risk patients for contrast-associated acute kidney injury (CA-AKI) helps to take early preventive interventions. The current study aimed to establish and validate an online pre-procedural nomogram for CA-AKI in patients undergoing coronary angiography (CAG).

**Methods:**

In this retrospective dataset, 4,295 patients undergoing CAG were enrolled and randomized into the training or testing dataset with a split ratio of 8:2. Optimal predictors for CA-AKI were determined by Least Absolute Shrinkage and Selection Operator (LASSO) and Random Forest (RF) algorithm. Nomogram was developed and deployed online. The discrimination and accuracy of the nomogram were evaluated by receiver operating characteristic (ROC) and calibration analysis, respectively. Clinical usefulness was estimated by decision curve analysis (DCA) and clinical impact curve (CIC).

**Results:**

A total of 755 patients (17.1%) was diagnosed with CA-AKI. 7 pre-procedural predictors were identified and integrated into the nomogram, including age, gender, hemoglobin, N-terminal of the prohormone brain natriuretic peptide, neutrophil-to-lymphocyte ratio, cardiac troponin I, and loop diuretics use. The ROC analyses showed that the nomogram had a good discrimination performance for CA-AKI in the training dataset (area under the curve, AUC = 0.766, 95%CI [0.737 to 0.794]) and testing dataset (AUC = 0.737, 95%CI [0.693 to 0.780]). The nomogram was also well-calibrated in both the training dataset (*P* = 0.965) and the testing dataset (*P* = 0.789). Good clinical usefulness was identified by DCA and CIC. Finally, this model was deployed in a web server for public use (https://duanbin-li.shinyapps.io/DynNomapp/).

**Conclusion:**

An easy-to-use pre-procedural nomogram for predicting CA-AKI was established and validated in patients undergoing CAG, which was also deployed online.

## Introduction

Coronary artery disease (CAD) is one of the main burdens in human diseases worldwide (1). With advances in interventional techniques, coronary angiography (CAG) and percutaneous coronary intervention (PCI) have contributed to a good prognosis in patients with CAD (2). However, increased administration of contrast agents makes contrast-associated acute kidney injury (CA-AKI) a new challenge (3, 4).

Contrast-associated acute kidney injury is a common complication after CAG or PCI and the third cause of iatrogenic renal dysfunction (5). The European Society of Urogenital Radiology (ESUR) defines CA-AKI as an increase in serum creatinine (Scr) ≥44 μmol/L (0.5 mg/dL) or ≥25% within 72 h after intravascular administration of iodinated contrast agents (6, 7). A meta-analysis of 29 studies found that the incidence of CA-AKI is about 5 to 20% in patients receiving intravascular contrast injections (8). Suffering from CA-AKI leads to increased subsequent mortality and contributes to a poor clinical prognosis (7, 9). In clinical practice, some protective interventions before procedure such as intravascular volume expansion, acetylcysteine use, and statins use, can reduce the incidence of CA-AKI (10). It will be beneficial to identify high-risk patients as early as possible and take necessary preventive interventions.

A series of risk-stratification models have been established and validated to identify high-risk CA-AKI patients (11). These models are highly predictive by integrating various well-established risk factors for CA-AKI (12, 13). Mehran risk score is the one of most commonly used prediction models for CA-AKI, which incorporates hypotension, intra-aortic balloon pump, congestive heart failure, age, anemia, diabetes, contrast agent volume, and estimated glomerular filtration rate (eGFR) (14). However, there are caveats in the clinical use of these models due to the undetermined predictor before CAG/PCI, such as the volume of contrast consumption, use of hemodynamic support devices, and PCI treatment (10). These undetermined predictors hinder the clinical use of risk-stratification models before CAG/PCI.

Pre-procedural preventive interventions may benefit high-risk CA-AKI patients. However, there is no risk-stratification model to identify these patients before CAG/PCI procedural. Therefore, we aimed to establish and validate a pre-procedural nomogram for the prediction of CA-AKI.

## Materials and Methods

### Study Population

**FIGURE 1:**
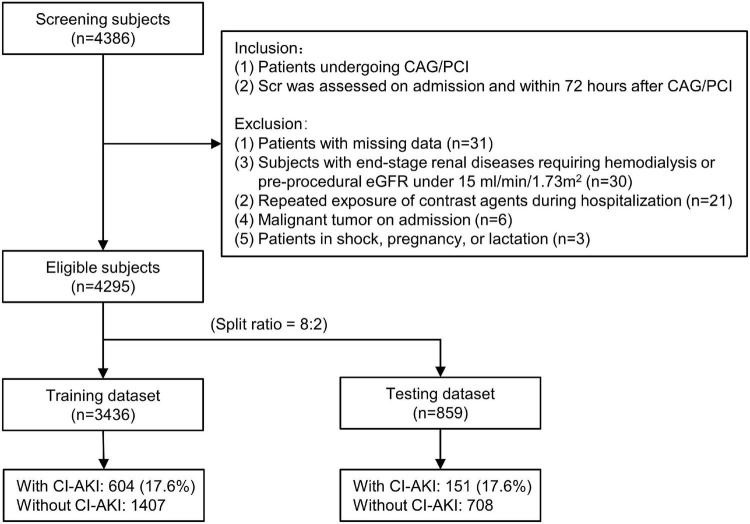
The flow chart of the current study. CAG indicates coronary angiography; PCI, percutaneous coronary intervention; CA-AKI, contrast-associated acute kidney injury; Scr, serum creatinine; eGFR, estimated glomerular filtration rate.

In the current retrospective dataset, consecutive patients undergoing CAG/PCI were eligible for screening from January 2009 to December 2019 at Sir Run Run Shaw Hospital and its medical consortium hospitals. Figure 1 shows the flow chart of the patient selection. The enrolled subjects were required to meet all of the following inclusion criteria: (1) patients undergoing CAG/PCI; (2) Scr levels were assessed on admission and within 72 h after CAG/PCI; Subjects were excluded if they met any of the following criteria: (1) patients with missing data (*n* = 31); (2) subjects with end-stage renal diseases requiring hemodialysis or pre-procedural eGFR under 15 ml/min/1.73 m^2^ (*n* = 30); (3) repeated exposure of contrast agents during hospitalization (*n* = 21); (4) active malignant tumor on admission (*n* = 6); (5) patients in shock, pregnancy, or lactation (*n* = 3). Eventually, a total of 4,295 subjects were included. Given a relatively large overall sample size, these subjects were randomized into the training dataset (*n* = 3436) and the testing dataset (*n* = 859) with a split ratio of 8:2. The training and testing dataset were used to establish and validate the predictive nomogram, respectively.

Transparent Reporting of a multivariable prediction model for Individual Prognosis Or Diagnosis (TRIPOD) guideline was followed to report this study (15). This study complied with the Declaration of Helsinki and was approved by the Medical Ethical Review Committee of Sir Run Run Shaw Hospital (No. 20201217 - 36).

### Study Predictors

Baseline variables includes traditional demographic data and candidate predictors for CA-AKI, which were based on previous literature, biologic plausibility, and/or consensus from clinical experience (7). In addition, the availability of variables is also considered, making the predictive model extensively applicable. Demographic variables included age, gender, diabetes, left ventricular ejection fraction (LVEF), hypertension, smoking, drinking, and mean arterial pressure. Laboratory testing variables included N-terminal of the prohormone brain natriuretic peptide (NT-proBNP), C-reactive protein (CRP), hemoglobin, total cholesterol, cardiac troponin I (cTnI), neutrophil-to-lymphocyte ratio (NLR), glycated hemoglobin A1c (HbA1c), and eGFR. Medication data included pre-procedural administration of statins, loop diuretics, and angiotensin receptor blocker (ARB).

Predictors were defined according to the standard criteria. Diabetes mellitus was defined as fasting plasma glucose ≥7.0 mmol/L, 2 h plasma glucose ≥11.1 mmol/L during oral glucose tolerance test, HbA1c ≥6.5% or a documented history of diabetes (16). Hypertension was defined as blood pressure ≥140/90 mmHg, or a documented history of hypertension. Mean arterial pressure was estimated by pre-procedural average blood pressure and computed by the following formula: mean arterial pressure (mmHg) = (systolic blood pressure + 2 × diastolic blood pressure)/3 (17). Current smoker was defined as having smoked any cigarettes on one or more of the 30 days preceding the admission (18). Current drinker was defined as having any alcohol consumption within last 30 days preceding the admission (19). LVEF was measured on admission by an experienced echocardiographer using Simpson’s biplane method (20). The cut-off point of abnormal NT-proBNP was age-adjusted (> 300 pg/ml in those <45 years, >900 pg/ml in those 45–70 years, and >1800 pg/ml in those >70 years) (21). Statin use was defined as regular use of pre-procedural statins for at least 8 weeks. The use of loop diuretic and angiotensin receptor blocker was defined as any pre-procedural medication use within 3 days before CAG/PCI. Chronic Kidney Disease Epidemiology Collaboration (CKD-EPI) formula was used to calculate eGFR (22).

### Primary Outcome

The primary outcome was the occurrence of CA-AKI after intravascular contrast injection. Scr levels were routinely assessed on admission and within 72 h after the contrast exposure. CA-AKI was determined by the elevation of Scr levels according to the diagnostic criteria of ESUR, including (1) an increase in Scr by more than 44 μmol/L (0.5 mg/dl) or 25%; (2) within 72 h of intravascular contrast injection; (3) no alternative etiology (6, 7).

### Predictor Selection

Significant predictors were selected by Least Absolute Shrinkage and Selection Operator (LASSO) and Random Forest (RF) algorithm in the training dataset. For LASSO algorithm, tenfold cross-validation was conducted to estimate the weight of the LASSO penalty, represented by λ. The indicator λ determines the complexity of the model: if λ = 0, no effect on regression parameters, if λ→∞, regression parameters will shrink and eventually set to 0 (23). A λ with the minimal cross-validation error was adopted and several non-zero estimations were eventually selected by LASSO. For RF algorithm, 500 trees and fivefold cross-validation was applied to determine the optimal number of trees under minimum cross-validation error. The mean decrease in Gini explains the importance of each predictor in the model with optimal tree number (24).

### Statistical Analysis

The continuous variables were represented by median (interquartile range) and compared by Mann–Whitney U test. The categorical variables were represented by count (proportion) and compared using chi-square test or Fisher’s exact test. LASSO and RF algorithms were employed to determine optimal predictors by R package “glmnet” and “randomForest,” respectively. Intersective predictors of both algorithms were eventually assembled into a binary logistic regression model (25). Nomogram was developed and visualized by R package “regplot.” Furthermore, the accuracy of the nomogram was estimated by calibration plots, and a likelihood ratio statistic (unreliability U index) was given to test the null hypothesis of calibration that intercept = 0, slope = 1 (26). The unreliability U index has a χ2 distribution with two degrees of freedom and was originally proposed by Cox (27). Receiver operating characteristic (ROC) curves were performed to verify the discrimination performance in the training and the testing dataset. The clinical usefulness of the nomogram was assessed by decision curve analysis (DCA) and clinical impact curve (CIC) across clinically relevant threshold probabilities. Two-sided *P*-value <0.05 were considered statistically significant. All statistical analyses were performed using SPSS software version 23.0 (SPSS Inc., Chicago, IL, United States) and R version 3.5.1 (The R Foundation for Statistical Computing, Vienna, Austria).

## Results

### Clinical Characteristics

In total, 4,295 patients undergoing CAG or PCI were included, and 755 (17.6%) patients were diagnosed with CA-AKI. Subjects were randomized into the training dataset (3,436 patients) and the testing dataset (859 patients) according to the split ratio of 8:2. Supplementary Table 1 displays the patient characteristics according to the dataset. The difference in CA-AKI prevalence between datasets was not significant (training *vs.* testing: 17.6% vs. 17.6%, *P* = 1.000). Table 1 shows the bivariate analyses according to CA-AKI in the training dataset. Patients who suffered CA-AKI were older (7 [7, 8] vs. 7 [6, 7] × 10 years, *P* < 0.001), had fewer males (60.1% vs. 67.9%, *P* < 0.001), and more diabetes (27.0% vs. 23.1%, *P* = 0.045). Additionally, CA-AKI patients had a relatively poor cardiac function (higher prevalence of abnormal NT-proBNP and lower LVEF levels), up-regulated inflammatory levels (higher CRP and NLR), and medication characteristics (more loop diuretics, fewer statins, and ARB) (all *P*-values <0.05).

**TABLE 1:** Summary of predictive variables according to CA-AKI in the testing dataset.

	Training dataset		
Predictive variables	without CA-AKI	CA-AKI	*P*-value
	*n* = 2832	*n* = 604	
**Demographic characteristics**			
Age, per 10 years	7 [6, 7]	7 [7, 8]	<0.001*
Male	1924 (67.9)	363 (60.1)	<0.001*
Diabetes	653 (23.1)	163 (27.0)	0.045*
Hypertension	1766 (62.4)	387 (64.1)	0.459
Current smoker	489 (17.3)	93 (15.4)	0.282
Current drinker	456 (16.1)	76 (12.6)	0.030*
Abnormal NT-proBNP	856 (30.2)	330 (54.6)	<0.001*
LVEF <50%	504 (17.8)	162 (26.8)	<0.001*
MAP ≥90 mmHg	1094 (38.6)	153 (25.3)	<0.001*
MAP <70 mmHg	34 (1.2)	20 (3.3)	<0.001*
Prior PCI	726 (25.6)	128 (21.2)	0.022*
Prior MI	221 (7.8)	45 (7.5)	0.768
Prior CABG	46 (1.6)	14 (2.3)	0.237
**Laboratory testing**			
Cardiac troponin I ≥0.02 ng/ml	962 (34.0)	362 (59.9)	<0.001*
Total cholesterol, mmol/L			0.031*
<3.0	439 (15.5)	120 (19.9)	
3.0–5.7	2144 (75.7)	437 (72.4)	
>5.7	249 (8.8)	47 (7.8)	
C-reactive protein ≥6 mg/L	755 (26.7)	255 (42.2)	<0.001*
NLR ≥5	599 (21.2)	255 (42.2)	<0.001*
Hemoglobin, g/L			<0.001*
≥115	2392 (84.5)	408 (67.5)	
90–114	381 (13.5)	147 (24.3)	
<90	59 (2.1)	49 (8.1)	
HbA1c ≥6.5%	853 (30.1)	233 (38.6)	<0.001*
eGFR <60 mL/min/1.73 m^2^	480 (16.9)	149 (24.7)	<0.001*
**Pre-procedural medication**			
Loop diuretics	763 (26.9)	325 (53.8)	<0.001*
Statins	2406 (85.0)	455 (75.3)	<0.001*
Angiotensin receptor blockers	891 (31.5)	146 (24.2)	<0.001*

Data are median [interquartile range] or n (%). NT-proBNP indicates N-terminal of the prohormone brain natriuretic peptide; LVEF, left ventricular ejection fraction; MAP, mean arterial pressure; PCI, percutaneous coronary intervention; MI, myocardial infarction; CABG, coronary artery bypass grafting; NLR, neutrophil-to-lymphocyte ratio; eGFR, estimated glomerular filtration rate; CA-AKI, contrast-associated acute kidney injury. *P < 0.05.

### Predictor Selection and Nomogram Development

**FIGURE 2:**
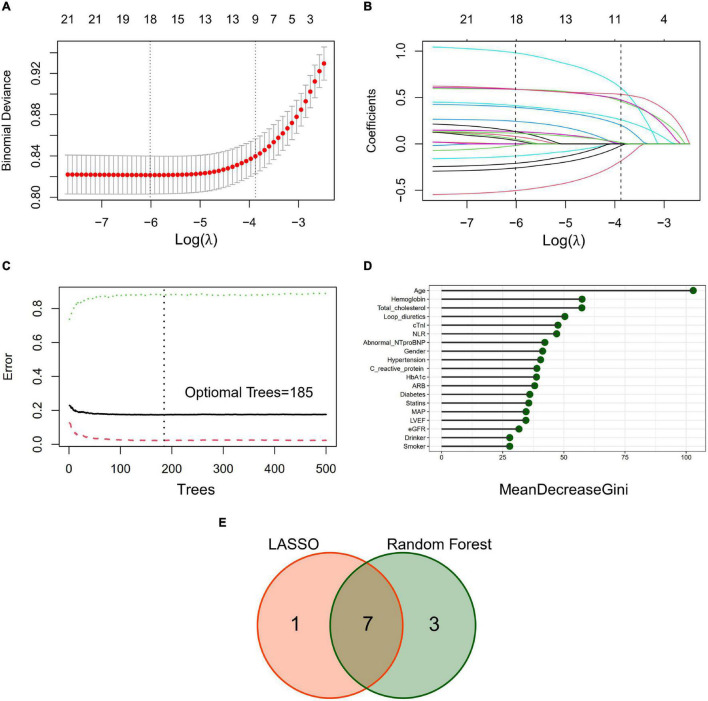
Predictive variables selection by the least absolute shrinkage and selection operator (LASSO) algorithm and random forest (RF) algorithm. **(A)** Identification of the optimal penalization estimate of lambda. Tenfold cross-validation with a minimum error criterion was used to determine the optimal penalization estimate of lambda in the LASSO regression. **(B)** The LASSO estimate profile of predictive variables. The left vertical line indicates the optimal lambda location, and the right vertical line indicates 1 standard error of optimal lambda. **(C)** Error convergence curve of the random forest model (500 trees). Optimal number of trees (*n* = 185) was determined according to the minimal error rate of fivefold cross-validation. **(D)** Importance ranking of candidate predictors in the random forest model. Mean decrease Gini index was calculated to define the importance of candidate predictors. **(E)** Venn diagram to determine identical predictors from LASSO and RF algorithm. A total of 7 identical predictors were determined from LASSO algorithm (8 variables) and RF algorithm (top 10 variables).

**FIGURE 3:**
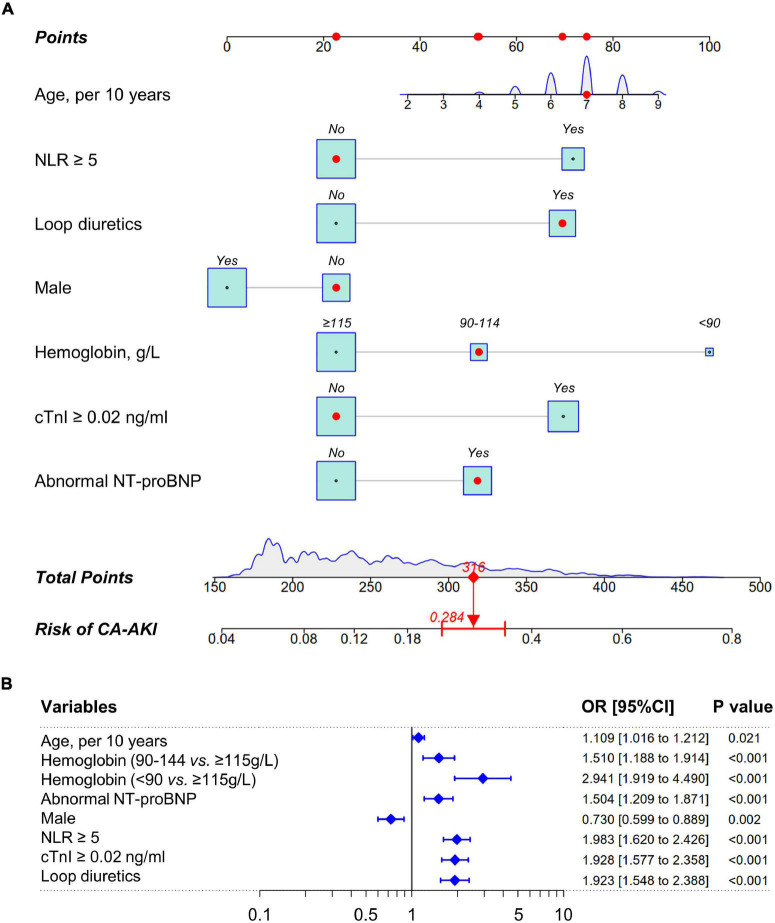
Nomogram to estimate the probability of CA-AKI. **(A)** A nomogram of CA-AKI established by pre-procedural routine data. The point of each predictive variable is determined by the corresponding location at the uppermost scale. Total points are the sum of each predictor. Total points of each patient indicate the corresponding probability of suffering CA-AKI after CAG/PCI procedural. The density plot or the box size for each predictor reflect population distribution in training dataset. The red marker depicts how to use this nomogram. **(B)** The forest plot of nomogram predictors. CRP indicates C-reactive protein; cTnI, cardiac troponin I; NT-proBNP, N-terminal of the prohormone brain natriuretic peptide; NLR, neutrophil-to-lymphocyte ratio; CA-AKI, contrast-associated acute kidney injury.

Least absolute shrinkage and selection operator algorithm was performed to determine a set of optimal predictors for CA-AKI (Figures 2A,B). A total of eight predictive variables were determined by LASSO regression, including age (per 10 years), hemoglobin (< 90, 90–114, ≥115 g/L), abnormal NT-proBNP (yes/no), male (yes/no), NLR ≥5 (yes/no), cTnI ≥0.02 ng/ml (yes/no), statins use (yes/no), and loop diuretics use (yes/no). RF algorithm with fivefold cross-validation achieves a stable cross-validation error by constructing 185 decision trees (Figure 2C). Under this RF model, the relative importance of candidate predictors for CA-AKI was ranked by mean decrease in Gini (Figure 2D). Eventually, a total of seven identical predictors were determined from LASSO algorithm (8 variables) and RF algorithm (top 10 variables), which was also visualized by the Venn diagram (Figure 2E). Selected predictors for CA-AKI by both algorithms were highly consistent. Further, these factors were assembled into a predictive model and visualized by a nomogram in the training dataset (Figure 3A). Selected predictors for CA-AKI by both algorithms are highly consistent. The estimate of each predictor was shown in the forest plot (Figure 3B). An online version of the dynamic nomogram was also deployed in the web server for public use.^1^

### Nomogram Validation by Discrimination Assessment

**FIGURE 4:**
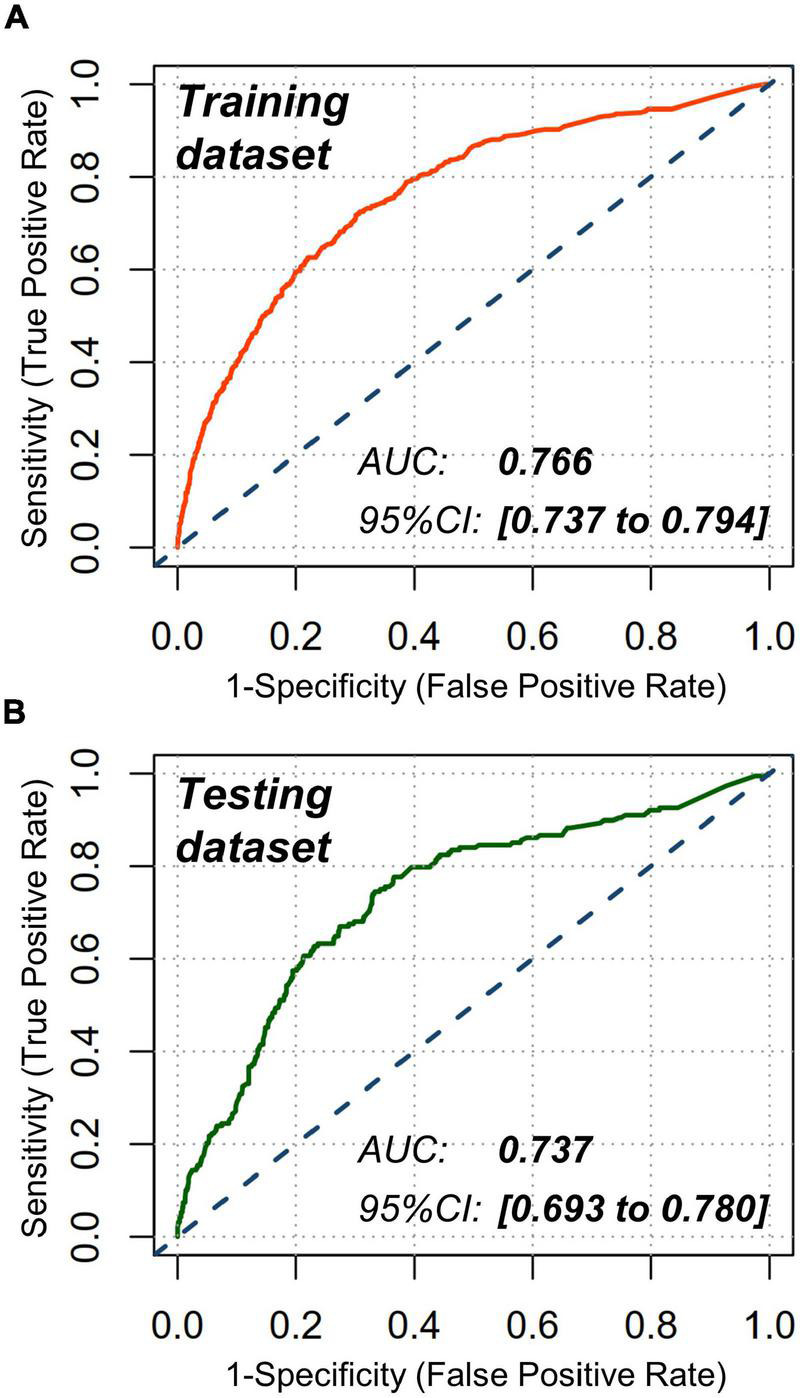
Receiver operating characteristic (ROC) analyses. Nomogram showed a good discrimination for CA-AKI in **(A)** training dataset (AUC: 0.766; 95% CI: 0.737 to 0.794) and **(B)** testing dataset (AUC: 0.737; 95% CI: 0.693 to 0.780). AUC indicates area under the curve; CI, confidence interval.

Receiver operating characteristic analyses were conducted to estimate the discrimination power of this nomogram with an area under the curve (AUC) being computed. Furthermore, a good discrimination power was verified in training dataset (AUC with 95% CI: 0.766 [0.737 to 0.794]) and testing dataset (AUC with 95% CI: 0.737 [0.693 to 0.780]), respectively (Figure 4).

### Nomogram Validation by Calibration Assessment

**FIGURE 5:**
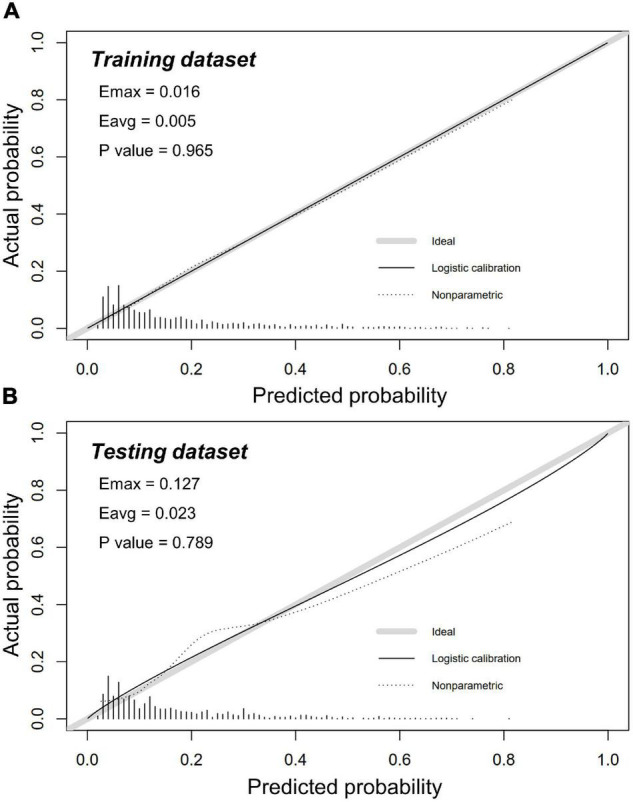
Calibration plots in training and testing dataset. Calibration plots showed excellent accuracy of the absolute risk prediction in panel **(A)** the training dataset (*P* = 0.965) and **(B)** the testing dataset (*P* = 0.789). If the nomogram has a good calibration, a 45-degree diagonal line will be presented between the actual rate of CA-AKI (*y*-axis) and the predicted probability of CA-AKI (*x*-axis). *P* > 0.05 indicates a good calibration with no difference between the actual rate and predicted probabilities.

Calibration of risk prediction was estimated by using the unreliability U test in datasets. An excellent concordance was identified between predicted and observed probabilities in the training dataset (*P* = 0.965) and the testing dataset (*P* = 0.789) (Figure 5). Conformity errors between predicted and observed probabilities were also calculated in the training dataset (average error = 0.005, maximal error = 0.016) and the testing dataset (average error = 0.023, maximal error = 0.127) (Figure 5).

### Nomogram Validation by Clinical Usefulness Assessment

**FIGURE 6:**
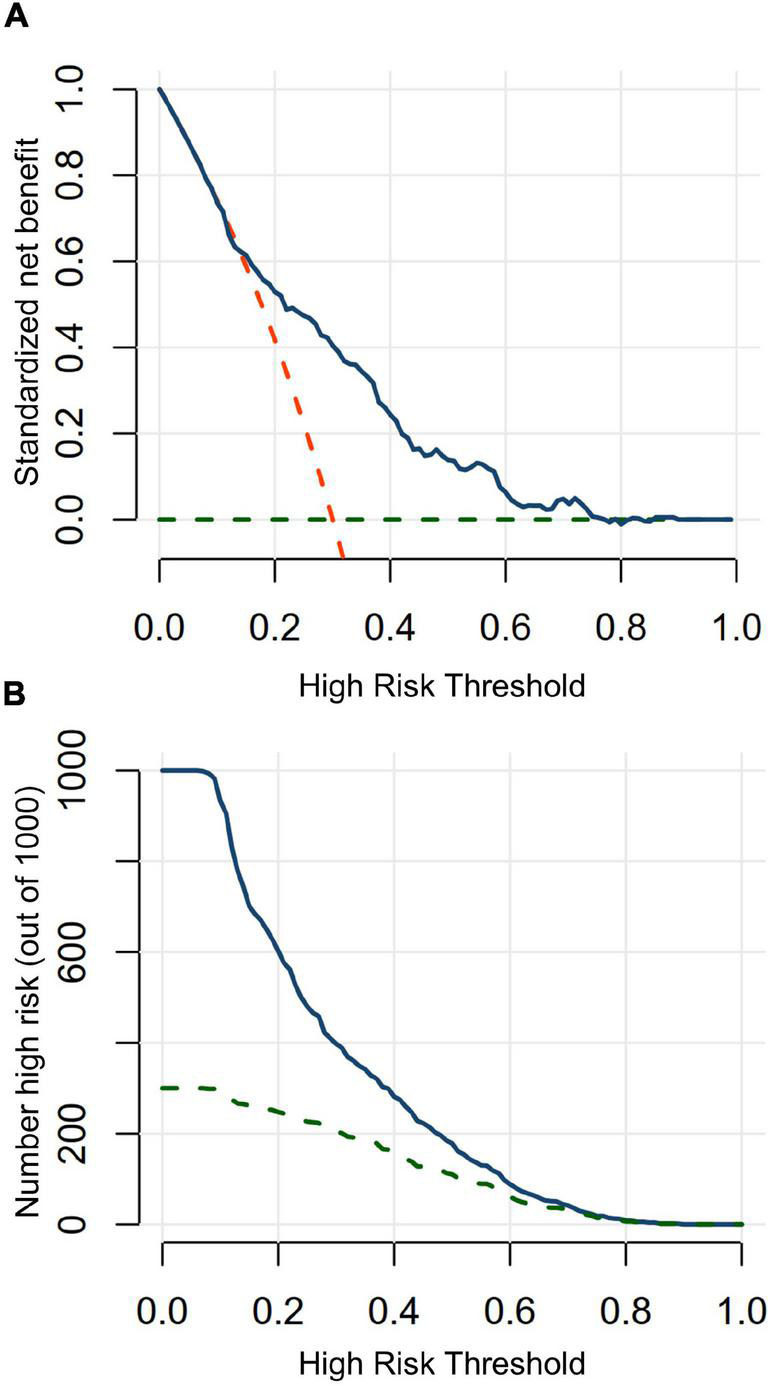
Decision curve analysis and clinical impact curve in testing in testing dataset. **(A)** Decision curve analysis. When a threshold probability is ranged from 15 to 80%, using the nomogram to predict CA-AKI achieves more benefits than the treat-all-patients scheme or the treat-none scheme. Blue solid line indicates the nomogram; red dashed line, the assumption that all patients have CA-AKI; green dashed line, the assumption that no patients have CA-AKI. **(B)** Clinical impact curve. Clinical impact curves analysis predicted the probability stratification of 1,000 subjects by using resample bootstrap method. The number of high-risk patients identified by nomogram (blue solid line) and high-risk patients with CA-AKI occurrence (green dashed line) were depicted in the plot under each threshold probability.

The DCA and CIC were performed to assess the clinical usefulness. The DCA curve suggests that the nomogram contributes the net benefit in almost all threshold probabilities, especially the range from 0.15 to 0.80 (Figure 6A). When the threshold probability <0.15, the net benefits of the nomogram were not superior to predict all patients suffering from CA-AKI. When the threshold probability ≥0.80, net benefits of the nomogram were not superior to predict no patients suffering from CA-AKI. The CIC of the nomogram depicted the predicted number of CA-AKI patients and true positive patients at different threshold probabilities (Figure 6B). The nomogram shows good clinical usefulness.

## Discussion

In this retrospective observational dataset, an online pre-procedural nomogram for CA-AKI was established and validated in patients undergoing CAG. This nomogram was validated to be well-calibrated and well-discriminated with seven easily available predictors incorporated. Good clinical usefulness was also verified by DCA and CIC. This nomogram enables physicians to identify high-risk CA-AKI patients before CAG/PCI and optimize clinical decision making.

Contrast-associated acute kidney injury has been the common complication after CAG or PCI and can lead to a poor prognosis (7). Some pre-procedural preventive interventions can reduce the incidence of CA-AKI, including intravascular volume expansion, acetylcysteine use, and statins use (10, 28, 29). Intensive preventive interventions may benefit high-risk patients of CA-AKI, which makes it imperative to identify these patients as early as possible (10). The Mehran risk score is one of the most commonly used predictive models for CA-AKI, which incorporates hypotension, intra-aortic balloon pump, congestive heart failure, age, anemia, diabetes, contrast agent volume, and eGFR (14). In comparison, our predictive model also incorporates several analogous predictors such as age, hemoglobin, and abnormal NT-proBNP. However, for general applicability, variables concerning CAG/PCI were not screened for our nomogram. Besides, Zhou et al also developed a CA-AKI nomogram for patients with acute myocardial infarction, incorporating age, hemoglobin, log (NT-proBNP), contrast volume, hypotension, and eGFR (30). Due to the contrast volume incorporated, this nomogram is also not applicable before CAG/PCI. However, there are still some variables that are analogous to our nomogram, such as age, hemoglobin, and NT-proBNP.

Recently, Mehran and colleagues developed and validated two CA-AKI predictive models by using pre-procedural variables alone and additional procedural variables, respectively (31). Both CA-AKI models showed similarly high discrimination in the validation cohort (C-statistic, pre-procedural variables only: 0.84; pre-procedural plus procedural variables: 0.86), indicating the rationality of using pre-procedural predictors alone. Despite differences in variables selection (current study: LASSO and random forest; Mehran’s model: forward stepwise selection), the current model includes some of the consistent predictors with Mehran’s model, such as age, hemoglobin levels, the maker of heart dysfunction (abnormal NT-proBNP), and the marker of myocardial infarction (cardiac TnI). Different from the current study, Mehran et al additionally presented the clinical relevance of the association between CA-AKI and all-cause mortality. However, some advantages of the current study are also worth noting. The current prediction model may have more predictive performance in the Chinese population due to specific data sources. In addition, the current predictive model is deployed on the web, which makes the predictive model highly accessible and easy to use.

In the current nomogram, seven easily available predictors were determined and assembled into the nomogram concerning demographic characteristics (age and gender), laboratory testing (hemoglobin, NLR, cTnI, and NT-proBNP), pre-procedural medication (loop diuretics). Demographic characteristics are correlated to the incidence of CA-AKI. Age has been assembled into numerous CA-AKI nomograms with advanced age predicting increased CA-AKI incidence (30, 32). This may be due to the prevalence of renal dysfunction in patients with advanced age. For predictor gender, Barbieri et al. found that female was more likely to suffer CA-AKI after PCI (33). Consistently, in the current study, the predictor gender was selected by algorithms, in which the female suggested a greater incidence of CA-AKI.

Laboratory testing data can also be helpful in predicting CA-AKI. Elevated NLR represents an up-regulated level of inflammation, which is correlated with an increased incidence of CA-AKI (34). Both NT-proBNP and cTnI were the focus of cardiovascular risk makers. Elevated NT-proBNP levels were identified to increase risks of CA-AKI and its subsequent mortality (35). Increased cTnI indicates myocardial injury or even myocardial infarction. The substudy of Harmonizing Outcomes With Revascularization and Stents in Acute Myocardial Infarction (HORIZONS-AMI) found that patients with acute myocardial infarction had a higher incidence of CA-AKI (36). As risk factors of CA-AKI, these indicators have been assembled into the current nomogram. Hemoglobin is a component of red blood cells, which could combine and transport oxygen to body tissues. Decreased hemoglobin has been identified as a crucial risk factor for CA-AKI (37). Consistent with Mehran risk score, hemoglobin was selected and integrated into the current nomogram (14). On the one hand, anemia decreases the oxygen delivery and exacerbates the oxidative stress in renal tubules cells, which decreases renal function and thus increases the CA-AKI risk (38, 39). On the other hand, renal diseases could decrease erythropoietin levels, which thus reduces hemoglobin production (40). Anemia may reflect an underlying renal dysfunction, which can increase CA-AKI risk.

Pre-procedural medications could also be underlying predictors for CA-AKI. For loop diuretics, several randomized controlled trials have demonstrated that pre-procedural administration of loop diuretics aggravated the risk of CA-AKI (41, 42). Besides, in clinical practice, loop diuretics administration is associated with the presence of cardiac dysfunction, which is also the risk factor for CA-AKI. Consistently, pre-procedural administration of loop diuretics was selected by algorithms and assembled in the nomogram.

Pre-procedural renal dysfunction has been an acknowledged risk factor of CA-AKI (10). In the training dataset, the baseline characteristics indicate that the CA-AKI group has a higher proportion of renal dysfunction (eGFR <60 mL/min/1.73 m^2^: 24.7% *vs.* 16.9%, *P* < 0.001). However, eGFR has not been determined as the final predictor by selection algorithms. Some reasons may be relevant. On the one hand, eGFR is estimated by the CKD-EPI equation, which includes age, gender, and Scr (22). Age and gender have been selected as the independent predictors for CA-AKI in this nomogram, which may partially replace the predictive power of eGFR. On the other hand, the proportion of renal dysfunction is relatively low, 17.7% of patients have eGFR <60 mL/min/1.73 m^2^ in the overall population. However, in the recent study by Mehran et al., this proportion was around 30%, almost double the current study (31). A better baseline renal function of the current study may reduce the discrimination performance of eGFR in predicting CA-AKI. In addition, current study excluded patients with eGFR <15 mL/min/1.73 m^2^ or end-stage renal diseases requiring hemodialysis, which were highly discriminative markers for predicting CA-AKI. The exclusion may further reduce the discrimination power of eGFR for CA - AKI.

Compared to previous CA-AKI nomograms, the current one was developed and validated in a larger study population, which is critical for the reliability of the predictive model. Besides, the current nomogram was established entirely using the pre-procedural predictors, which improved the applicability of this nomogram. By CA-AKI prediction before CAG/PCI, this nomogram makes it easy for physicians to identify high-risk patients and individualize preventive interventions. Early identification of high-risk patients can help clinicians decide whether to use multiple preventive strategies for high-risk patients. In addition, clinicians will be alerted to consider the nephrotoxicity of the prescribed drugs, minimize the dose of contrast agents used in CAG or PCI, and use the RenalGuard system to promote rapid excretion of contrast agents.

However, there were several study limitations worthy of mention. On the one hand, the inherent selection bias and external validation absence may reduce the generalization performance of the predictive model. The generalization ability needs to be further estimated in more external validation datasets. On the other hand, a total of 19 screening predictors were pre-determined according to accessibility and underlying clinical significance from demographic data, laboratory data, and medication data. Therefore, selection bias of screening predictors is inevitable. Despite its limitations, the current nomogram is still an easy-to-use online tool to predict CA-AKI before CAG/PCI.

## Conclusion

An easy-to-use pre-procedural nomogram for predicting CA-AKI was established and validated in patients undergoing CAG, which was also deployed online. Pre-procedural prediction will facilitate the clinical decision-making on preventive interventions.

## Data Availability Statement

The raw data supporting the conclusions of this article will be made available by the authors, without undue reservation.

## Ethics Statement

The studies involving human participants were reviewed and approved by the Ethics Committee of Sir Run Run Shaw Hospital of Zhejiang University. The ethics committee waived the requirement of written informed consent for participation.

## Author Contributions

WZ and DWL conceived and designed the study. DBL organized these data and drafted the manuscript with the help of XY, HJ, ML, and MG. ZC and DL analyzed the data. HJ drew the pictures. WZ, DWL, and GF detected any errors in the whole process. All authors have read and approved the manuscript for submission.

## Conflict of Interest

The authors declare that the research was conducted in the absence of any commercial or financial relationships that could be construed as a potential conflict of interest.

## Publisher’s Note

All claims expressed in this article are solely those of the authors and do not necessarily represent those of their affiliated organizations, or those of the publisher, the editors and the reviewers. Any product that may be evaluated in this article, or claim that may be made by its manufacturer, is not guaranteed or endorsed by the publisher.

## Footnotes

1
https://duanbin-li.shinyapps.io/DynNomapp/

## Abbreviations

CAD: coronary artery disease
CAG: coronary angiography
PCI: percutaneous coronary intervention
CA-AKI: contrast-associated acute kidney injury
ESUR: European Society of Urogenital Radiology
Scr: serum creatinine
eGFR: estimated glomerular filtration rate
LVEF: left ventricular ejection fraction
NT-proBNP: N-terminal of the prohormone brain natriuretic peptide
cTnI: cardiac troponin I
CRP: C-reactive protein
NLR: neutrophil-to-lymphocyte ratio
HbA1c: glycated hemoglobin A1c
ARB: angiotensin receptor blocker
CKD-EPI: chronic kidney disease epidemiology collaboration
LASSO: least absolute shrinkage and selection operator
RF: random forest
ROC: receiver operating characteristic
DCA: decision curve analysis
CIC: clinical impact curve
AUC: area under the curve.
