# Supplementary material for: An Online Pre-procedural Nomogram for the Prediction of Contrast-Associated Acute Kidney Injury in Patients Undergoing Coronary Angiography
Source: Front Med (Lausanne). 2022 Mar 11;9:839856. doi: 10.3389/fmed.2022.839856 (PMC8961873; doi:10.3389/fmed.2022.839856)
Supplement: Supplementary file 1 [file Table_1.pdf]

Supplementary Material

Table S1. Summary of predictive variables according to the dataset

Predictive variables	Overall n=4295	Training dataset n=3436	Testing dataset n=859	*P* value
**Demographic characteristics**				
Age, per 10 years	7 [6, 7]	7 [6, 7]	7 [6, 7]	0.250
Male	2827 (65.8)	2287 (66.6)	540 (62.9)	0.044
Diabetes	1012 (23.6)	816 (23.7)	196 (22.8)	0.590
Hypertension	2706 (63.0)	2153 (62.7)	553 (64.4)	0.364
Current smoker	728 (16.9)	582 (16.9)	146 (17.0)	0.959
Current drinker	671 (15.6)	532 (15.5)	139 (16.2)	0.636
Abnormal NT-proBNP	1477 (34.4)	1186 (34.5)	291 (33.9)	0.748
LVEF <50 %	818 (19.0)	666 (19.4)	152 (17.7)	0.285
MAP ≥90 mmHg	1555 (36.2)	1247 (36.3)	308 (35.9)	0.843
**Laboratory testing**				
Cardiac troponin I ≥0.02 ng/ml	1637 (38.1)	1324 (38.5)	313 (36.4)	0.272
Total cholesterol, mmol/L				0.728
< 3.0	689 (16.0)	559 (16.3)	130 (15.1)	
3.0-5.7	3236 (75.3)	2581 (75.1)	655 (76.3)	
> 5.7	370 (8.6)	296 (8.6)	74 (8.6)	
C-reactive protein ≥6 mg/L	1233 (28.7)	1010 (29.4)	223 (26.0)	0.048
NLR ≥5	1065 (24.8)	854 (24.9)	211 (24.6)	0.895
Hemoglobin, g/L				0.925
≥115	3499 (81.5)	2800 (81.5)	699 (81.4)	
90-114	663 (15.4)	528 (15.4)	135 (15.7)	
<90	133 (3.1)	108 (3.1)	25 (2.9)	
HbA1c ≥6.5 %	1357 (31.6)	1086 (31.6)	271 (31.5)	1.000
eGFR <60 mL/min/1.73m^2	759 (17.7)	629 (18.3)	130 (15.1)	0.031
**Pre-procedural medication**				
Loop diuretics	1361 (31.7)	1088 (31.7)	273 (31.8)	0.967
Statins	3573 (83.2)	2861 (83.3)	712 (82.9)	0.799
Angiotensin receptor blockers	1295 (30.2)	1037 (30.2)	258 (30.0)	0.967
**Primary outcome**				
CA-AKI	755 (17.6)	604 (17.6)	151 (17.6)	1.000

Data are median [interquartile range] or n (%). NT-proBNP indicates N-terminal of the prohormone brain natriuretic peptide; LVEF, left ventricular ejection fraction; MAP, mean arterial pressure; NLR, neutrophil-to-lymphocyte ratio; eGFR, estimated glomerular filtration rate; CA-AKI, contrast-associated acute kidney injury.
